# Endoplasmic Reticulum Stress-Related Signature for Predicting Prognosis and Immune Features in Hepatocellular Carcinoma

**DOI:** 10.1155/2022/1366508

**Published:** 2022-08-14

**Authors:** Genhao Zhang, Jianping Sun

**Affiliations:** ^1^Department of Blood Transfusion, The First Affiliated Hospital of Zhengzhou University, Zhengzhou, China; ^2^Department of Pathology, Zhengzhou YIHE Hospital, Zhengzhou, China

## Abstract

Hepatocellular carcinoma (HCC) with cancer cells under endoplasmic reticulum (ER) stress has a poor prognosis. This study is aimed at discovering credible biomarkers for predicting the prognosis of HCC based on ER stress-related genes (ERSRGs). We constructed a novel four-ERSRG prognostic risk model, including PON1, AGR2, SSR2, and TMCC1, through a series of bioinformatic approaches, which can accurately predict survival outcomes in HCC patients. Higher risk scores were linked to later grade, recurrence, advanced TNM stage, later T stage, and HBV infection. In addition, 20 fresh frozen tumors and normal tissues from HCC patients were collected and used to validate the genes expressed in the signature by qRT-PCR and immunohistochemical (IHC) assays. Moreover, we found the ER stress-related signature could reflect the infiltration levels of different immune cells in the tumor microenvironment (TME) and forecast the efficacy of immune checkpoint inhibitor (ICI) treatment. Finally, we created a nomogram incorporating this ER stress-related signature. In conclusion, our constructed four-gene risk model associated with ER stress can accurately predict survival outcomes in HCC patients, and the model's risk score is associated with the poor clinical classification.

## 1. Introduction

The endoplasmic reticulum (ER) is a multifunctional organelle consisting of branching tubes and flattened vesicles that are the main site of protein synthesis and transport, lipid biosynthesis, and calcium storage [1, 2]. However, many factors, including inhibition of protein glycosylation, oxidative stress, nutritional deficiencies, imbalance of calcium homeostasis, and hypoxia, could reduce the efficiency of ER in processing protein folding and finally lead to ER stress and unfolded protein response (UPR) [3, 4]. UPR plays a crucial role in regulating cellular adaptation to ER stress by increasing ER content, improving ER protein folding capacity, and downgrading misfolded proteins [5, 6]. Three transmembrane ER sensors, including activating transcription factor 6 (ATF6), protein kinase R- (PKR-) like endoplasmic reticulum kinase (PERK), and inositol-requiring enzyme 1 (IRE1*α*), have been found to determine the triggering of ER stress and subsequent activation of the UPR [7]. With the increasing recognition of ER stress mechanisms, ER stress dysregulation has been found to play an essential role in various human diseases, including cardiometabolic diseases [8–10], diabetes [11, 12], chronic kidney disease [13, 14], Alzheimer's disease [15, 16], and cancers [17–20]. Chronic activation of the UPR caused by ER stress, viral infection, or hepatic obesity may lead to liver dysfunction and disturbances in lipid and glucose metabolism [21]. ER stress plays a crucial role in the pathogenesis of the nonalcoholic fatty liver disease (NAFLD) [22] and is strongly associated with survival and death in HCC patients [23]. Recent studies suggested that ER stress and UPR have emerged as new signaling targets for therapeutic interventions in NAFLD and HCC [3, 24–26]. In the current study, a novel prognostic risk model based on ER stress-related genes (ERSRGs) was constructed, which could be effectively used for prognostic classification of HCC patients and utilized as a potential target for individualized immunotherapy.

## 2. Materials and Methods

### 2.1. Public Datasets and Generation of ERSRGs

This study included mRNA expression data and clinical features of HCC patients from three publicly available datasets including TCGA-LIHC, ICGC (LIRI-JP), and GSE14520. ERSRGs were available from previous research [4]. The general clinical characteristics of HCC patients are shown in Table S1.

### 2.2. Molecular Subtype Identification by Nonnegative Matrix Factorization (NMF) Algorithm

The 365 HCC samples in TCGA were grouped using the NMF algorithm with the criteria “brunet” and 50 iterations based on ERSRGs. The number of clusters (*K*) ranged from 2 to 6, with cophenetic, dispersion, and profile being used to determine the ideal number of clusters. Kaplan-Meier survival analysis was also undertaken to see if there were any changes in survival across the NMF subtypes.

### 2.3. Prognostic Risk Score Model Construction and Functional Analysis

The univariable Cox relapses were to begin with performed to calculate the affiliation between ERSRGs and survival results of HCC patients in three cohorts. LASSO-Cox relapse strategy and stepwise Cox relapse examination were at that point performed to survey the overcovering prognosis-related qualities and set up prognostic characteristics. Risk score was at last set up based on the premise of directly combining the equation underneath with the mRNA expression level duplicated the multivariate Cox relapse coefficient (*β*) demonstrate. Risk score = (*β*mRNA1 × mRNA1) + (*β*mRNA2 × mRNA2) + ⋯+(*β*mRNAn × mRNAn). We stratified patients in TCGA dataset into two subgroups due to the ideal hazard score edge. The prescient control and autonomy of the prognostic signature were evaluated by receiver operating characteristic (ROC) curve, Kaplan-Meier survival examination, and Cox relative risk relapse investigation. Gene set enrichment analysis (GSEA) between the two subgroups was performed to distinguish the altogether cautioned GO items with FDR < 0.05.

### 2.4. Quantitative Real-Time PCR (qRT-PCR) and Immunohistochemical (IHC) Analysis

Twenty fresh-frozen tumors and normal tissues from HCC patients who underwent liver tissue resection were collected, and all patients signed informed consent. All pathological data were evaluated and codiagnosed by two independent pathologists. All methods were performed following relevant guidelines and regulations. qRT-PCR was used to detect the mRNA levels of genes in the model [27]. Primer sequences are shown in Table S2. IHC assays were used to explore the protein expression of SSR2 in normal and HCC tissues. SSR2 antibody was obtained from Proteintech (China). Two pathologists independently assessed the results.

### 2.5. Immune Status Calculation and Immune Infiltrate Analysis

The immune status of each sample was assessed by applying the ESTIMATE algorithm to the TCGA cohort and calculating immune and stromal scores. The association between risk scores and immune and stromal scores was analyzed by Pearson correlation analysis. To explore the impacts of the prognostic model on immunotherapies, we calculated the relationship between risk score and 15 potentially available targeted immune checkpoint genes [28]. Furthermore, to assess the potential association between prognostic signature and tumor-infiltrating immune cells (TIICs) in the HCC microenvironment, the TCGA database was used to measure the abundance ratios of TIICs through CIBERSORT [29] (http://cibersort.stanford.edu/), Timer [30], Quantiseq [31], and xCell [32].

### 2.6. Genetic Alterations and TMB Analysis

The mutation and CNA data of 350 HCC patients were downloaded from TCGA to analyze the difference of genetic alterations between the high- and low-risk score subgroups with R package “maftools,” and the tumor mutation burden (TMB) of each patient was subsequently assessed.

### 2.7. Drug Susceptibility Analysis

The association between anticancer drug sensitivity and mRNA molecules in our risk model was directly explored in the CellMiner database [33]. 574 in advanced clinical trials and 216 Food and Drug Administration- (FDA-) approved drugs were used for follow-up analyses. Drugs with adjusted *P* value < 0.001 and Pearson correlation coefficient > 0.3 as cut-off criteria were considered tumor-sensitive drugs.

### 2.8. Statistical Analysis

Categorical data were compared with the Pearson chi-square test or Fisher exact test whenever appropriate, and quantitative variables were analyzed using the independent-samples *t*-test. ROC curve analysis and Kaplan-Meier survival analysis were performed to assess the prediction performance of survival outcomes with R software (Version 4.0.3). Cox proportional model was performed to analyze the relationship between prognostic signature and survival outcomes, together with other clinical features. Results were considered statistically significant when the *P* value < 0.05.

## 3. Results

### 3.1. Molecular Subtype Identification Based on ERSRGs

**Figure 1:**
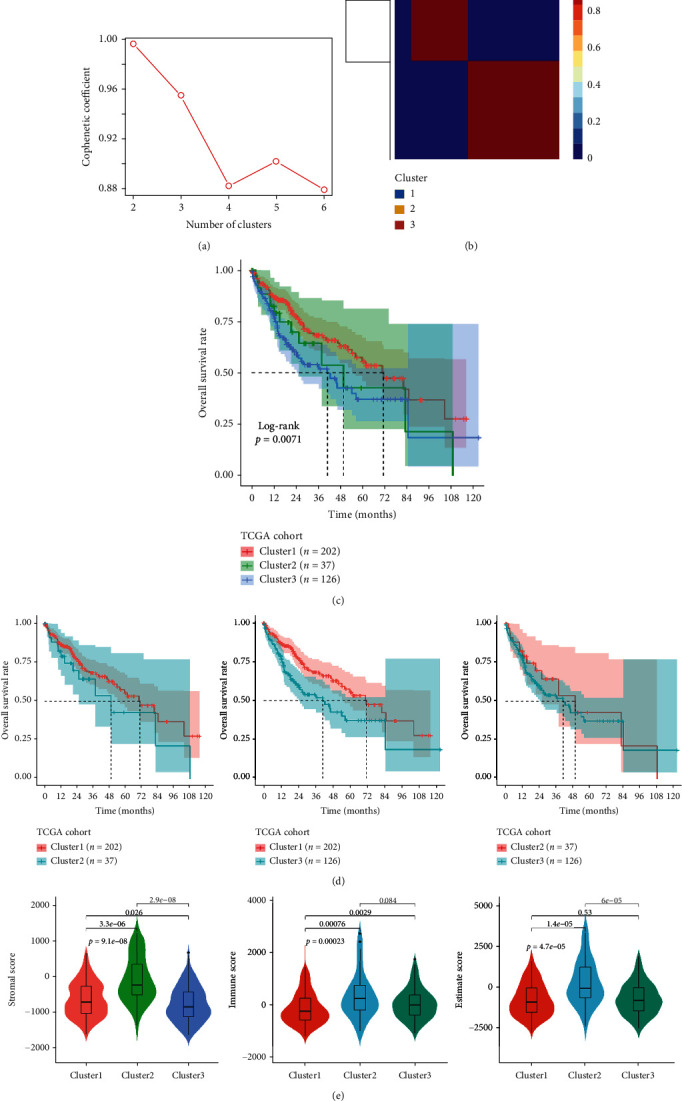
Molecular subtypes identification based on ERSRGs. (a) The optimal number of clusters was identified as three based on the cophenetic. (b) 365 HCC samples were separated into three subcategories. (c) Substantial differences were found between patients in the three groupings. (d) Two-by-two comparisons were made between the three groups. (e) Stromalscore, immunescore, and ESTIMATEscore were different among the three clusters.

By using the NMF algorithm, the optimal number of clusters was identified as three based on the cophenetic (Figure 1(a)). Then, 365 HCC samples were separated into three subcategories based on the ERSRGs (Figure 1(b)), and substantial differences were found between patients in the three groupings (Figure 1(c)). When two-by-two comparisons were made between the three groups, only cluster 1 and cluster 3 were significantly different from each other (Figure 1(d)). Interestingly, the stromalscore, immunescore, and ESTIMATEscore were different among the three clusters (Figure 1(e)).

### 3.2. Identification of Overlapped ERSRGs in the Three Cohorts

**Figure 2:**
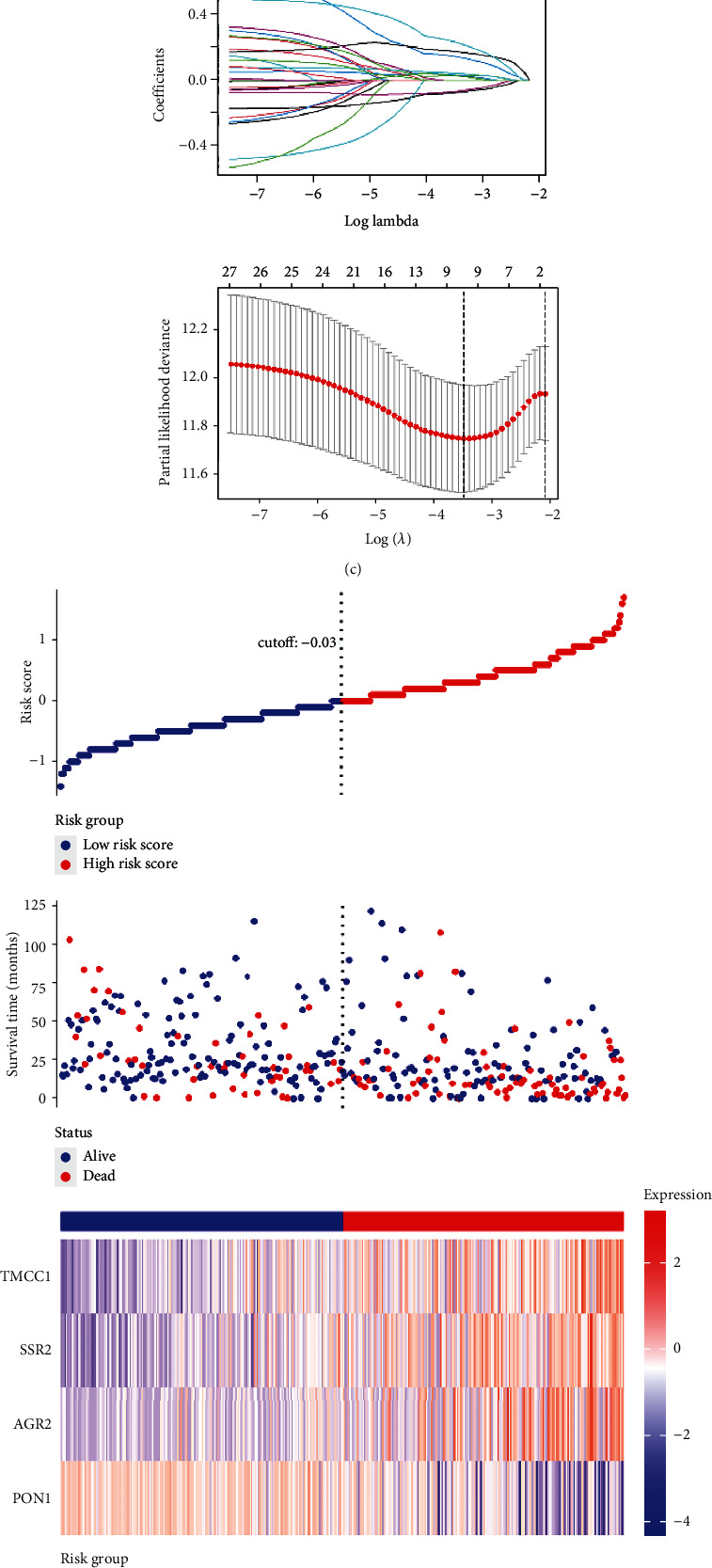
Development and survival examinations of four-gene signature in TCGA. (a) Identification of overlapping prognostic ERSRGs in TCGA, ICGC, and GSE14520 datasets. (b) Enrichment analysis of 28 overlapping prognostic ERSRGs. (c) The parameter selection in the LASSO-Cox analysis was adjusted by 10 cross-validations. (d) Distribution of risk scores, OS status, and gene expression profiles. (e) Kaplan-Meier survival plot. (f) Characteristics of predicted 1-, 2-, and 3-year OS rates in ROC analysis.

As calculated by univariable Cox regressions with an adjusted *P* value < 0.05, 329 ERSRGs in TCGA, 225 ERSRGs in ICGC, and 152 ERSRGs in GSE14520 cohort had significant prognostic relevance, respectively (Figure 2(a)). Then, 28 overlapped ERSRGs are mainly enriched in ER stress-related biological processes and are used for further analysis (Figure 2(b)).

### 3.3. Establishment of an ER Stress-Related Signature in TCGA

**Figure 3:**
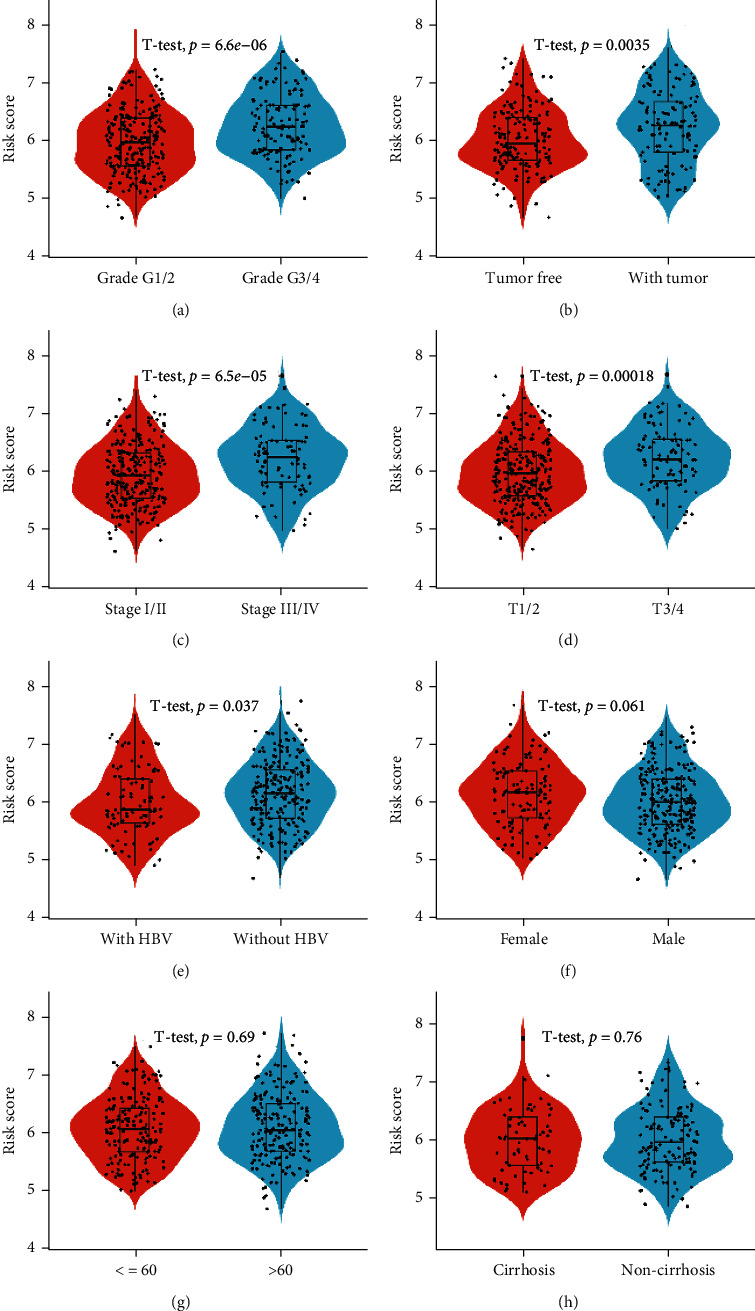
Higher risk scores were linked to later grade (a), recurrence (b), advanced TNM stage (c), later T stage (d), and HBV infection (e). As for age, gender, and cirrhosis status, no significant differences were found (f–h).

Overlapped ERSRGs were selected by performing the LASSO-Cox regression model based on the minimum value of *λ*, and 9 genes were screened as shown in Figure 2(c). These genes were then placed into a stepwise Cox proportional model, and finally, a prognostic four-gene signature was identified. Risk score = (0.07012933 × AGR2) + (0.42892985 × SSR2)–(0.12903062 × PON1) + (0.40769605 × TMCC1). Only SSR2 showed differential expression in normal and tumor tissues (Figure S1(a)), although all four genes had prognostic significance (Figure S1(b)). The expression of the four genes in HCC cell lines was shown in Figure S1(c). Risk scores for HCC patients were calculated with the above formula, and patients were stratified into high- or low-risk subgroups with an optimal risk score threshold (Figure 2(d)). Kaplan-Meier survival analysis revealed that patients with higher risk scores were significantly relevant to poorer survival outcomes (Figure 2(e)), and ROC analysis revealed that this signature had a good prognostic performance with AUCs at 1-, 2-, and 3-year of 0.769, 0.738, and 0.715 (Figure 2(f)). The association between risk score and clinical characteristics including age, gender, grade, stage, and vascular invasion and the value of AFP, cirrhosis, HBV infection status, and tumor status were evaluated. The results revealed that higher risk scores were linked to later grade (Figure 3(a)), recurrence (Figure 3(b)), advanced TNM stage (Figure 3(c)), later T stage (Figure 3(d)), and HBV infection (Figure 3(e)). As for age, gender, and cirrhosis status, no significant differences were found (Figures 3(f)–3(h)). In addition, further stratified survival analysis was applied for different clinical characteristics, and the results demonstrated that this prognostic model could further differentiate patients with different clinical characteristics including age, vascular invasion, grade, recurrence, TNM stage, gender, HBV infection status, and AF*P* value (Supplementary Figure S2). Finally, the results of GO and KEGG functional analysis of the differential genes in the high- and low-risk score groups are shown in Supplementary Figure S3. To explore whether the four-gene signature could be acted as an independent prognostic model for HCC patients, univariable and multivariate Cox analyses were performed, and results revealed that this signature could be served as an independent prognostic factor for HCC patients (HR = 2.203, 95% CI 1.313-3.694, *P* < 0.001) in TCGA.

### 3.4. Verification of the Signature in the ICGC and GSE14520 Cohorts

**Figure 4:**
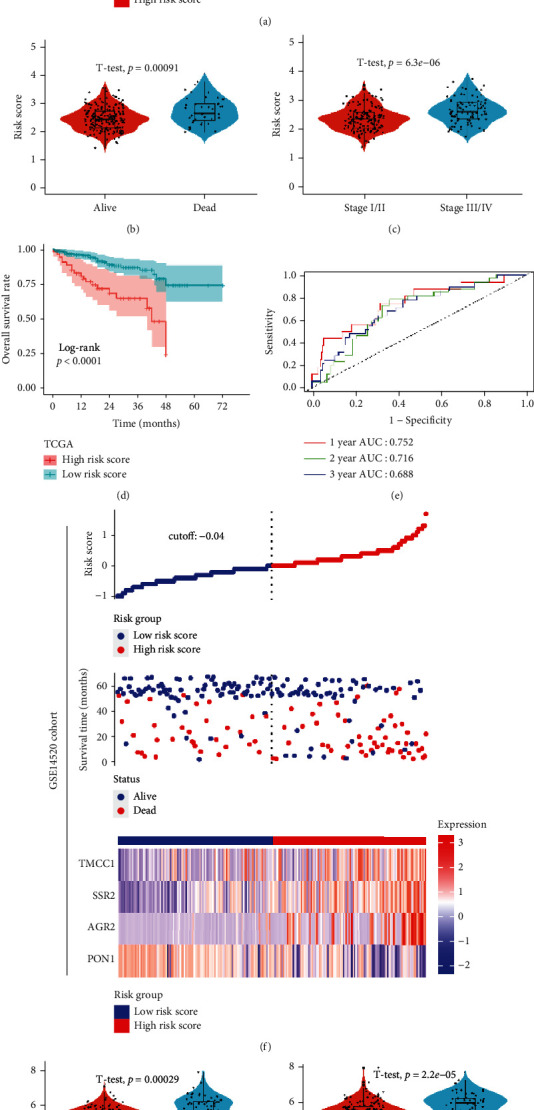
Validation of this feature in ICGC and GSE14520 cohorts. (a) Distribution of risk scores, OS status, and gene expression profiles in ICGC cohort. (b) Comparison of risk scores among patients with different survival statuses. (c) Comparison of risk scores among patients with different TNM stages. (d) Kaplan-Meier survival plot. (e) Characteristics of predicted 1-, 2-, and 3-year OS rates in ROC analysis. (f) Distribution of risk scores, OS status, and gene expression profiles in the GSE14520 cohort. (g) Comparison of risk scores among patients with different survival statuses. (h) Comparison of risk scores among patients with different TNM stages. (i) Kaplan-Meier survival plot. (j) Characteristics of predicted 1-, 2-, and 3-year OS rates in ROC analysis.

To validate the signature, ICGC and GSE14520 datasets were applied as validation cohorts. Risk scores of patients were calculated with the same formula, and patients were stratified into high- or low-risk subgroups in the ICGC (Figure 4(a)) and GSE14520 cohort (Figure 4(f)). We found lower scores in patients with HCC who were alive (Figure 4(b)) or at earlier TNM stages (Figure 4(c)) in the ICGC dataset. Kaplan-Meier survival analysis revealed that patients with higher risk scores were prominently relevant to poorer OS rates in the ICGC cohort (Figure 4(d)). ROC analysis revealed that this signature had a good prognostic performance with AUCs at 1-, 2-, and 3-year of 0.752, 0.716, and 0.698 in the ICGC cohort (Figure 4(e)). The same results were found in the GSE14520 dataset (Figures 4(g) and 4(h)). Finally, the results of univariable and multivariate Cox analyses revealed that this signature could be served as an independent prognostic factor for HCC patients (ICGC: HR = 2.749, 95% CI 1.424-5.310, *P* < 0.001; GSE14520: HR = 1.713, 95% CI 1.224-2.398, *P* < 0.001).

### 3.5. Genetic Alterations and TMB Analysis

**Figure 5:**
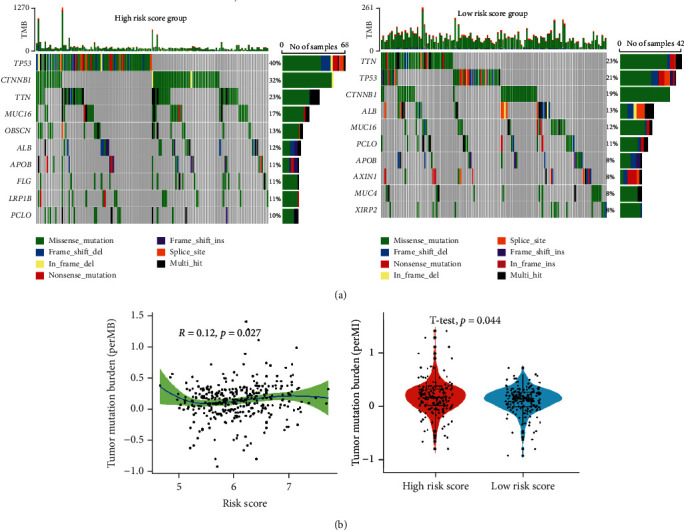
Genetic mutation and TMB analysis. (a) Tumor maps of mutated genes in two subgroups. (b) Correlation and differential analysis of risk scores and TMB.

The results of genetic alterations analysis indicated that the mutation rates of the top 10 most significantly mutated genes were remarkably different in the two subgroups (Figure 5(a)). Subsequently, the TMB of each patient was assessed. We found that the risk score was closely related to TMB, and patients in the high-risk scores subgroup had significantly increased TMB (Figure 5(b)).

### 3.6. Immune Infiltrates and Immune Checkpoint Gene Analysis

**Figure 6:**
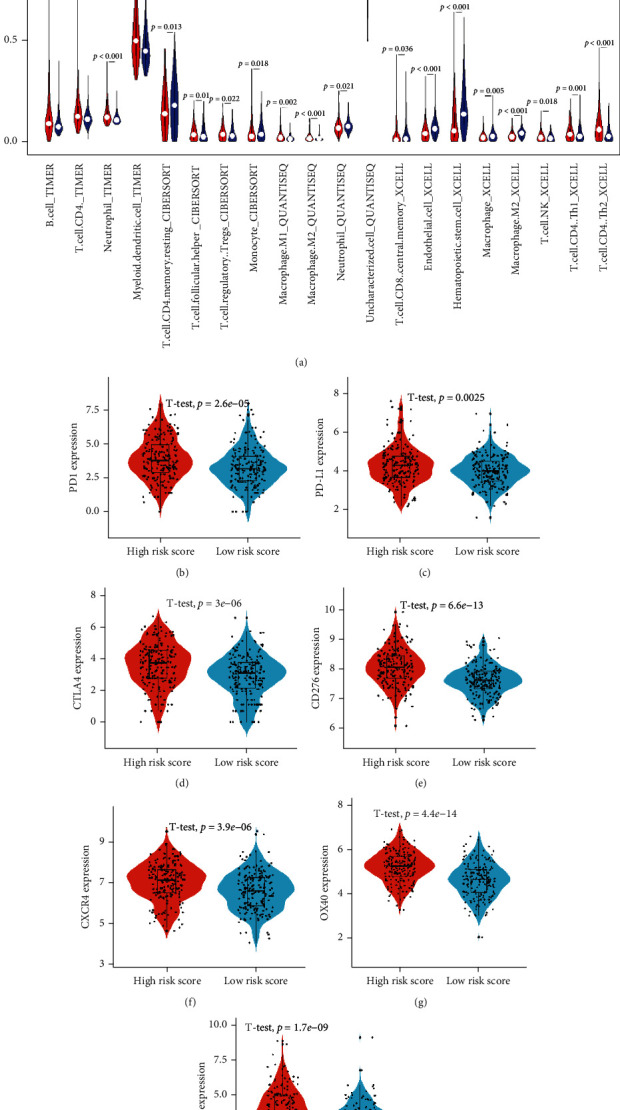
Immune infiltrates analysis. (a) Violin plot showing the abundance differentiation of TIICs in two subgroups. (b–h) Differential analysis of immune checkpoint genes in two subgroups.

As shown in Figure 6(a), according to the results of the Timer algorithm, HCC patients in the high-risk score subgroup had modestly increased ratios of B cells, CD4^+^ T cells, neutrophils, and myeloid dendritic cells. The results of the CIBERSORT demonstrated that HCC patients in the low-risk score subgroup had modestly increased ratios of resting CD4 memory cells and monocyte cells, while patients in the high-risk score subgroup had significantly elevated ratios of T helper cells and Treg cells. Moreover, the results of the Quantiseq demonstrated that HCC patients in the low-risk score subgroup had modestly increased ratios of neutrophil and uncharacterized cells, while patients in the high-risk score subgroup had significantly elevated ratios of macrophages M1 and M2 cells (Figure 4(a)). The results of the xCell demonstrated that HCC patients in the low-risk score subgroup had modestly increased ratios of CD8 central memory cells, endothelial cells, hematopoietic stem cells, macrophage cells, macrophage M2 cells, and NK cells, while patients in the high-risk score subgroup had significantly elevated ratios of CD4 Th1 and Th2 cells. In the following, we found that patients in the high-risk subgroup had significantly increased PD1, PD-L1, CD276, CTLA4, CXCR4, OX40, and CD137 (Figures 6(b)–6(h)), indicating that immune checkpoint inhibitor (ICIs) treatment was more effective for patients in high-risk subgroup.

### 3.7. Establishment of a Nomogram Model in TCGA

**Figure 7:**
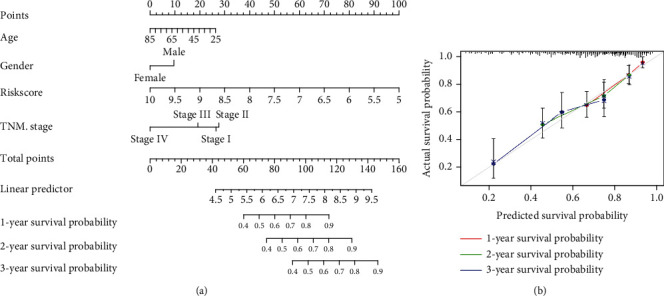
The predictive significance of the signatures was verified in the nomogram model. (a) Nomogram combining the four genetic signatures. (b) Calibration plots of 1-, 2-, and 3-year survival probabilities.

To investigate the coefficient prediction efficiency of this signature, a nomogram model was established in the TCGA dataset, and the result revealed that the nomogram with a C-index of 0.723 could help us provide a quantitative method for predicting the 1-, 2-, and 3-year survival rate accurately (Figure 7(a)). The overlap between the forecasted and actual probabilities of 1-, 2-, and 3-year survival rates in the calibration curves indicated good agreement (Figure 7(b)).

### 3.8. Comparison with the Previous Signatures

Comparing the prediction potential among several genetic signatures can help researchers learn more about their prognostic significance. When compared with other previous signatures [34–37], as shown in Figure S4, our four-gene signature provided the best survival prediction capacity with fewer genes.

### 3.9. Drug Susceptibility Analysis

Among the 574 in advanced clinical trials and 216 Food and Drug Administration- (FDA-) approved drugs, 55 were considered tumor-sensitive drugs (Table S3), and the top 16 most significant tumor-sensitive drugs were shown in Supplementary Figure S5.

### 3.10. Expression Levels of Genes in the Risk Model

**Figure 8:**
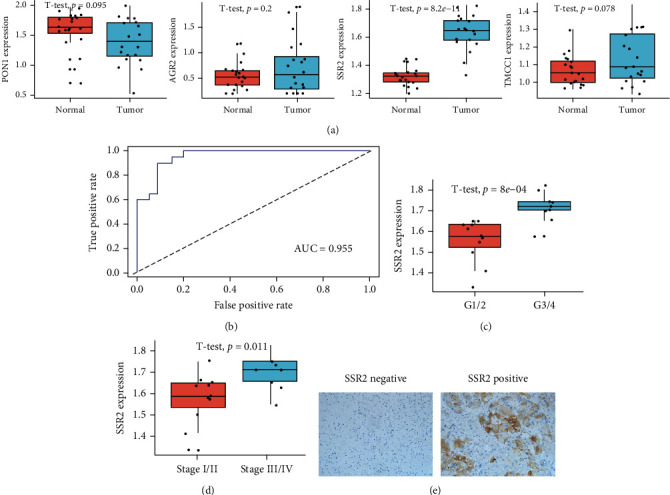
Validation of this feature in clinical samples. (a) Expression levels of mRNA molecules in our risk model. (b) ROC curve. (c, d) SSR2 was associated with poor progression. (e) The protein expression of SSR2 was overexpressed in HCC compared to normal tissues.

In keeping with the results of the GEPIA analysis [38], we found that only SSR2 was differentially expressed in tumor and normal samples by qRT-PCR (Figure 8(a)). Furthermore, after calculating the patients' risk scores using the same formula, we found that the scores could distinguish well between normal and tumor tissue (Figure 8(b)). Considering that only SSR2 was differentially expressed, we analyzed it further. We also found that the expression of SSR2 was associated with poor progression in HCC patients (Figures 8(c) and 8(d)). The protein expression of SSR2 was overexpressed in HCC tissues compared to normal tissues (Figure 8(e)). Finally, the results of univariable and multivariate Cox analyses revealed that SSR2 could be served as an independent prognostic factor for HCC patients (HR = 2.1.759, 95% CI 1.191-2.599, *P* = 0.004) in TCGA.

## 4. Discussion

There is growing evidence that ER stress-mediated cell proliferation, metabolic conversion, and genomic destabilization are important in the development of many chronic liver diseases, including alcoholic liver disease (ALD), hepatic fibrosis, NAFLD, HBV, HCV hepatitis, and HCC [39–41]. What is more, some synergistic effects between virus infection, alcohol abuse, NAFLD, and ER stress were found in the carcinogenesis of HCC [23]. In addition, ER stress can enhance cancer cell immune evasion and promote recurrence and metastasis by affecting the tumor microenvironment (TME) [42, 43]. ER stress-mediated UPR induces autophagy via IRE1*α*, PERK, and ATF6 signaling channels and stimulates vascular endothelial growth factor (VEGF) secretion by macrophages, thereby promoting vasculogenesis in TME [44, 45]. Studies to date have shown that ER stress plays a substantial role in regulating tumor cell fate through altered metabolic status and has emerged as a novel signaling target for the treatment of HCC. Inhibition of IRE1*α*, XBP1s, and PERK expression could trigger tumor cell death under ER stress conditions [24–26]. Proteasome inhibitor MLN2238 exacerbates ER stress and promotes cycle stagnation and apoptosis [46]. Sorafenib induces increased ER stress and activates cellular autophagy in HerpG2 cells [47]. Therefore, we hypothesized that aberrant expression of ER stress-related genes may have prognostic value for HCC patients.

In the current study, a novel four-gene prognostic risk model based on ERSRGs was constructed and exhibited superior accuracy in forecasting the survival outcomes and 1-, 2-, and 3-year survival rate of HCC patients in TCGA, ICGC, and GSE14520 cohorts. More importantly, this feature was an independent risk factor for HCC patients when other clinical factors in the three cohorts were taken into account. In addition, significant effects of this feature on the immune microenvironment of HCC and the response to immune checkpoint inhibitors were investigated. Patients in the high-risk score subgroup had significantly increased TMB values, PD1, PD-L1, CD276, CTLA4, CXCR4, OX40, and CD137, indicating that ER stress could affect the immune microenvironment in HCC, and immune checkpoint inhibitor (ICIs) treatment was more effective for patients in high-risk subgroup. In addition, we explored the association between the expression of genes in the risk model and anticancer drug sensitivity in the CellMiner database and identified 55 tumor-sensitive drugs that may be available for the treatment of HCC patients. Finally, to exploit the full potential of this risk model, a nomogram was constructed and exhibited superior predictive performance. Therefore, our four genetic risk models associated with ER stress can accurately predict survival outcomes in HCC patients and facilitate the selection of the best-individualized treatment.

Of the four genes in HCC patients, SSR2, TMCC1, and AGR2 expressions were positively correlated with poor prognosis, while PON1 expression was negatively correlated with poor prognosis. As an important member of the paraoxonase (PON) family, PON1 plays a very important role in protecting mammalian organisms from oxidative stress [48, 49]. Endothelial dysfunction in the body can be brought on by glycosylated PON1-inducing ER stress [50]. PON1 is also implicated in the apoptosis that ER stress causes in tumor cells [51] and serum PON1 level is a powerful prognostic factor and can be used to evaluate microvascular invasion in HCC [52]. An endoplasmic reticulum-based proto-oncogene called anterior gradient-2 (AGR2) is in charge of maintaining the ER homeostasis. High AGR2 expression improves cell folding and quality control, which are essential for the response to ER stress [53]. AGR2 is also involved in the development and progression of multiple tumors, including pancreatic [54], prostate [55], colorectal [56], breast [57], and endometrial [58] cancers. AGR2 may serve as a potential drug target to improve drug sensitivity during cancer treatment [59]. AGR2 also plays a very important role in the proliferation and metastasis of HCC cells [60]. Signal sequence receptor 2 (SSR2) is a protein involved in the unfolded protein response of the endoplasmic reticulum. Through the translocation of proteins necessary for URP, SSR2 may be particularly implicated in melanoma cells' resistance to ER stress [61]. Our study found that SSR2 is upregulated and is strongly associated with poor prognosis in HCC patients. Upregulated SSR2 could be involved in hepatocarcinogenesis and metastasis through the regulation of epithelial-mesenchymal transition (EMT) [62]. The transmembrane coiled-coil domain (TMCC1) is widely present in vertebrates and lower organisms and belongs to the representative members of the TMCC family. TMCC1 localizes to the rough ER through its C-terminal transmembrane domain and binds to ribosomal proteins through its cytoplasmic region, participating in the regulation of ER stress-associated proteins [63]. Although TMCC1 has not been reported in the literature to be associated with HCC, in our study, we found that HCC patients with high expression of TMCC1 had a poor prognosis. Of course, this requires later collection of multiple HCC cases with complete survival data to validate this result. Overall, the four genes involved in our prognostic model are all related to ER stress, and a more in-depth study of their mechanism of action in HCC may provide new ideas for immunotherapy targeting ER stress in the future.

Compared with the 7-gene prognostic model constructed in our previous study presented as a preprint [64] and other previous signatures [34–37], this 4-gene prognostic model predicted better with fewer genes. Of course, our research has certain limitations. This feature's effectiveness may be hampered by the diversity and individual heterogeneity of HCC patients. Furthermore, more in vivo and in vitro research is needed to further examine the expression and prognostic predictive relevance of these four genes at the protein level, as well as their unique processes in HCC.

## 5. Conclusions

In summary, we constructed an ERSRG-based risk model in this study, which can effectively classify HCC patients for prognostic prediction and individualized immunotherapy targeting ER stress.

## Data Availability

The data used to support the findings of this study are available from the corresponding authors upon request.

## Supplementary Materials

Supplementary Materials Figure S1: expression levels and prognostic values of the four genes in normal and HCC tissues explored in GEPIA (a, b) and CCLE database (c). Figure S2: this prognostic model could further differentiate patients with different clinical characteristics. Figure S3: the results of GO and KEGG functional analysis of the differential genes in the high- and low-risk score groups. Figure S4: comparison of the predictive power of the four-gene prognostic model with other prognostic models for survival. Figure S5: top 16 most important tumor-sensitive drugs. Table S1: clinical characteristics of HCC patients involved in the study. Table S2: the sequences of the qRT-PCR primers used in this study. Table S3: 55 tumor-sensitive drugs targeting tumor cell stemness.

## References

[1] Wang M., Kaufman R. J. (2016). Protein misfolding in the endoplasmic reticulum as a conduit to human disease. *Nature*.

[2] Schwarz D. S., Blower M. D. (2016). The endoplasmic reticulum: structure, function and response to cellular signaling. *Cellular and Molecular Life Sciences*.

[3] Yukimoto A., Watanabe T., Sunago K. (2021). The long noncoding RNA of RMRP is downregulated by PERK, which induces apoptosis in hepatocellular carcinoma cells. *Scientific Reports*.

[4] Zhang Q., Guan G., Cheng P., Cheng W., Yang L., Wu A. (2021). Characterization of an endoplasmic reticulum stress-related signature to evaluate immune features and predict prognosis in glioma. *Journal of Cellular and Molecular Medicine*.

[5] Kapuy O., Márton M., Bánhegyi G., Vinod P. K. (2020). Multiple system-level feedback loops control life-and-death decisions in endoplasmic reticulum stress. *FEBS Letters*.

[6] Yu J., Li T., Liu Y. (2020). Phosphorylation switches protein disulfide isomerase activity to maintain proteostasis and attenuate ER stress. *The EMBO Journal*.

[7] Ron D., Walter P. (2007). Signal integration in the endoplasmic reticulum unfolded protein response. *Nature Reviews Molecular Cell Biology*.

[8] Zhu H., Zhou H. (2021). Novel insight into the role of endoplasmic reticulum stress in the pathogenesis of myocardial ischemia-reperfusion injury. *Oxidative Medicine and Cellular Longevity*.

[9] Ajoolabady A., Wang S., Kroemer G. (2021). ER stress in cardiometabolic diseases: from molecular mechanisms to therapeutics. *Endocrine Reviews*.

[10] Kratochvílová H., Mráz M., Kasperová B. J. (2021). Different expression of mitochondrial and endoplasmic reticulum stress genes in epicardial adipose tissue depends on coronary atherosclerosis. *International Journal of Molecular Sciences*.

[11] Liu Z., Zhu H., He C. (2021). Nicorandil attenuates high glucose-induced insulin resistance by suppressing oxidative stress-mediated ER stress PERK signaling pathway. *BMJ Open Diabetes Research & Care*.

[12] Shrestha N., De Franco E., Arvan P., Cnop M. (2021). Pathological *β*-cell endoplasmic reticulum stress in type 2 diabetes: current evidence. *Front Endocrinol (Lausanne)*.

[13] Liu X., Chen A., Liang Q. (2021). Spermidine inhibits vascular calcification in chronic kidney disease through modulation of SIRT1 signaling pathway. *Aging Cell*.

[14] Wang Y. L., Lee Y. H., Hsu Y. H. (2021). The kidney-related effects of polystyrene microplastics on human kidney proximal tubular epithelial cells HK-2 and male C57BL/6 mice. *Environmental Health Perspectives*.

[15] Minaei A., Sarookhani M. R., Haghdoost-Yazdi H., Rajaei F. (2021). Hydrogen sulfide attenuates induction and prevents progress of the 6-hydroxydopamine-induced Parkinsonism in rat through activation of ATP-sensitive potassium channels and suppression of ER stress. *Toxicology and Applied Pharmacology*.

[16] Gupta P., Tiwari S., Singh A., Pal A., Mishra A., Singh S. (2021). Rivastigmine attenuates the Alzheimer's disease related protein degradation and apoptotic neuronal death signalling. *The Biochemical Journal*.

[17] Jiang Z. B., Xu C., Wang W. (2021). Plumbagin suppresses non-small cell lung cancer progression through downregulating ARF1 and by elevating CD8^+^T. *Pharmacological Research*.

[18] Jang H., Jun Y., Kim S. (2021). FCN3 functions as a tumor suppressor of lung adenocarcinoma through induction of endoplasmic reticulum stress. *Cell Death & Disease*.

[19] Chang C. Y., Pan P. H., Wu C. C. (2021). Endoplasmic reticulum stress contributes to gefitinib-induced apoptosis in glioma. *International Journal of Molecular Sciences*.

[20] Li C., Zhang K., Pan G. (2021). Dehydrodiisoeugenol inhibits colorectal cancer growth by endoplasmic reticulum stress-induced autophagic pathways. *Journal of Experimental & Clinical Cancer Research*.

[21] Wei J., Yuan Y., Chen L. (2018). ER-associated ubiquitin ligase HRD1 programs liver metabolism by targeting multiple metabolic enzymes. *Nature Communications*.

[22] Flessa C. M., Kyrou I., Nasiri-Ansari N. (2021). Endoplasmic reticulum stress and autophagy in the pathogenesis of non-alcoholic fatty liver disease (NAFLD): current evidence and perspectives. *Current Obesity Reports*.

[23] Wei J., Fang D. (2021). Endoplasmic reticulum stress signaling and the pathogenesis of hepatocarcinoma. *International Journal of Molecular Sciences*.

[24] Pavlović N., Calitz C., Thanapirom K. (2020). Inhibiting IRE1*α*-endonuclease activity decreases tumor burden in a mouse model for hepatocellular carcinoma. *eLife*.

[25] Wu S., Du R., Gao C., Kang J., Wen J., Sun T. (2018). The role of XBP1s in the metastasis and prognosis of hepatocellular carcinoma. *Biochemical and Biophysical Research Communications*.

[26] Vandewynckel Y. P., Laukens D., Bogaerts E. (2015). Modulation of the unfolded protein response impedes tumor cell adaptation to proteotoxic stress: a PERK for hepatocellular carcinoma therapy. *Hepatology International*.

[27] Zhang G. (2020). Expression and prognostic significance of BANF1 in triple-negative breast cancer. *Cancer Management and Research*.

[28] Yang C., Huang X., Liu Z., Qin W., Wang C. (2020). Metabolism-associated molecular classification of hepatocellular carcinoma. *Molecular Oncology*.

[29] Gentles A. J., Newman A. M., Liu C. L. (2015). The prognostic landscape of genes and infiltrating immune cells across human cancers. *Nature Medicine*.

[30] Li T., Fan J., Wang B. (2017). TIMER: a web server for comprehensive analysis of tumor-infiltrating immune cells. *Cancer Research*.

[31] Finotello F., Mayer C., Plattner C. (2019). Molecular and pharmacological modulators of the tumor immune contexture revealed by deconvolution of RNA-seq data. *Genome Medicine*.

[32] Aran D., Hu Z., Butte A. J. (2017). xCell: digitally portraying the tissue cellular heterogeneity landscape. *Genome Biology*.

[33] Reinhold W. C., Sunshine M., Liu H. (2012). CellMiner: a web-based suite of genomic and pharmacologic tools to explore transcript and drug patterns in the NCI-60 cell line set. *Cancer Research*.

[34] Liu G. M., Zeng H. D., Zhang C. Y., Xu J. W. (2019). Identification of a six-gene signature predicting overall survival for hepatocellular carcinoma. *Cancer Cell International*.

[35] Liang J. Y., Wang D. S., Lin H. C. (2020). A novel ferroptosis-related gene signature for overall survival prediction in patients with hepatocellular carcinoma. *International Journal of Biological Sciences*.

[36] Ouyang G., Yi B., Pan G., Chen X. (2020). A robust twelve-gene signature for prognosis prediction of hepatocellular carcinoma. *Cancer Cell International*.

[37] Zhang B. H., Yang J., Jiang L. (2020). Development and validation of a 14-gene signature for prognosis prediction in hepatocellular carcinoma. *Genomics*.

[38] Tang Z., Li C., Kang B., Gao G., Li C., Zhang Z. (2017). GEPIA: a web server for cancer and normal gene expression profiling and interactive analyses. *Nucleic Acids Research*.

[39] Baiceanu A., Mesdom P., Lagouge M., Foufelle F. (2016). Endoplasmic reticulum proteostasis in hepatic steatosis. *Nature Reviews. Endocrinology*.

[40] Lebeaupin C., Vallée D., Hazari Y., Hetz C., Chevet E., Bailly-Maitre B. (2018). Endoplasmic reticulum stress signalling and the pathogenesis of non-alcoholic fatty liver disease. *Journal of Hepatology*.

[41] Kim J. Y., Garcia-Carbonell R., Yamachika S. (2018). ER stress drives lipogenesis and steatohepatitis via caspase-2 activation of S1P. *Cell*.

[42] Urra H., Dufey E., Avril T., Chevet E., Hetz C. (2016). Endoplasmic reticulum stress and the hallmarks of cancer. *Trends Cancer*.

[43] Mahadevan N. R., Rodvold J., Sepulveda H., Rossi S., Drew A. F., Zanetti M. (2011). Transmission of endoplasmic reticulum stress and pro-inflammation from tumor cells to myeloid cells. *Proceedings of the National Academy of Sciences of the United States of America*.

[44] Xia S. W., Wang Z. M., Sun S. M. (2020). Endoplasmic reticulum stress and protein degradation in chronic liver disease. *Pharmacological Research*.

[45] Cullen S. J., Fatemie S., Ladiges W. (2013). Breast tumor cells primed by endoplasmic reticulum stress remodel macrophage phenotype. *American Journal of Cancer Research*.

[46] Augello G., Modica M., Azzolina A. (2018). Preclinical evaluation of antitumor activity of the proteasome inhibitor MLN2238 (ixazomib) in hepatocellular carcinoma cells. *Cell Death & Disease*.

[47] Zhou B., Lu Q., Liu J. (2019). Melatonin increases the sensitivity of hepatocellular carcinoma to sorafenib through the PERK-ATF4-Beclin1 pathway. *International Journal of Biological Sciences*.

[48] Shi S., Buck T. M., Nickerson A. J., Brodsky J. L., Kleyman T. R. (2022). Paraoxonase 2 is an ER chaperone that regulates the epithelial Na^+^ channel. *American Journal of Physiology Cell Physiology*.

[49] Lee S. J., Kang H. K., Choi Y. J. (2018). PEP-1-paraoxonase 1 fusion protein prevents cytokine-induced cell destruction and impaired insulin secretion in rat insulinoma cells. *BMB Reports*.

[50] Yu W., Liu X., Feng L. (2017). Glycation of paraoxonase 1 by high glucose instigates endoplasmic reticulum stress to induce endothelial dysfunction in vivo. *Scientific Reports*.

[51] Bacchetti T., Ferretti G., Sahebkar A. (2019). The role of paraoxonase in cancer. *Seminars in Cancer Biology*.

[52] Ding G. Y., Zhu X. D., Ji Y. (2020). Serum PON1 as a biomarker for the estimation of microvascular invasion in hepatocellular carcinoma. *Annals of Translational Medicine*.

[53] Dumartin L., Alrawashdeh W., Trabulo S. M. (2017). ER stress protein AGR2 precedes and is involved in the regulation of pancreatic cancer initiation. *Oncogene*.

[54] Hong X., Li Z. X., Hou J. (2021). Effects of ER-resident and secreted AGR2 on cell proliferation, migration, invasion, and survival in PANC-1 pancreatic cancer cells. *BMC Cancer*.

[55] Tang D., He J., Dai Y. (2022). Targeting KDM1B-dependent miR-215-AR-AGR2-axis promotes sensitivity to enzalutamide-resistant prostate cancer. *Cancer Gene Therapy*.

[56] Zhang H., Chi J., Hu J. (2021). Intracellular AGR2 transduces PGE2 stimuli to promote epithelial-mesenchymal transition and metastasis of colorectal cancer. *Cancer Letters*.

[57] Wang J., Huang K., Shi L., Zhang Q., Zhang S. (2020). CircPVT1 promoted the progression of breast cancer by regulating miR-29a-3p-mediated AGR2-HIF-1*α* pathway. *Cancer Management and Research*.

[58] Gong W., Ekmu B., Wang X., Lu Y., Wan L. (2020). AGR2-induced glucose metabolism facilitated the progression of endometrial carcinoma via enhancing the MUC1/HIF-1*α* pathway. *Human Cell*.

[59] Negi H., Merugu S. B., Mangukiya H. B. (2019). Anterior gradient-2 monoclonal antibody inhibits lung cancer growth and metastasis by upregulating p53 pathway and without exerting any toxicological effects: a preclinical study. *Cancer Letters*.

[60] Bian J., He L., Wu Y. (2020). Anterior gradient 2-derived peptide upregulates major histocompatibility complex class I-related chains A/B in hepatocellular carcinoma cells. *Life Sciences*.

[61] Garg B., Pathria G., Wagner C., Maurer M., Wagner S. N. (2016). Signal sequence receptor 2 is required for survival of human melanoma cells as part of an unfolded protein response to endoplasmic reticulum stress. *Mutagenesis*.

[62] Hong X., Luo H., Zhu G. (2020). SSR2 overexpression associates with tumorigenesis and metastasis of hepatocellular carcinoma through modulating EMT. *Journal of Cancer*.

[63] Zhang C., Kho Y. S., Wang Z. (2014). Transmembrane and coiled-coil domain family 1 is a novel protein of the endoplasmic reticulum. *PLoS One*.

[64] Zhang G. (2021). Endoplasmic reticulum stress-related classification for prognosis prediction in hepatocellular carcinoma. *Preprint (Version 1) Available at Research Square*.

