# Supplementary material for: Endoplasmic Reticulum Stress-Related Signature for Predicting Prognosis and Immune Features in Hepatocellular Carcinoma
Source: J Immunol Res. 2022 Aug 14;2022:1366508. doi: 10.1155/2022/1366508 (PMC9393196; doi:10.1155/2022/1366508)
Supplement: Supplementary Materials — Figure S1: expression levels and prognostic values of the four genes in normal and HCC tissues explored in GEPIA (a, b) and CCLE database (c). Figure S2: this prognostic model could further differentiate patients with different clinical characteristics. Figure S3: the results of GO and KEGG functional analysis of the differential genes in the high- and low-risk score groups. Figure S4: comparison of the predictive power of the four-gene prognostic model with other prognostic models for survival. Figure S5: top 16 most important tumor-sensitive drugs. Table S1: clinical characteristics of HCC patients involved in the study. Table S2: the sequences of the qRT-PCR primers used in this study. Table S3: 55 tumor-sensitive drugs targeting tumor cell stemness. [file 1366508.f1.docx]

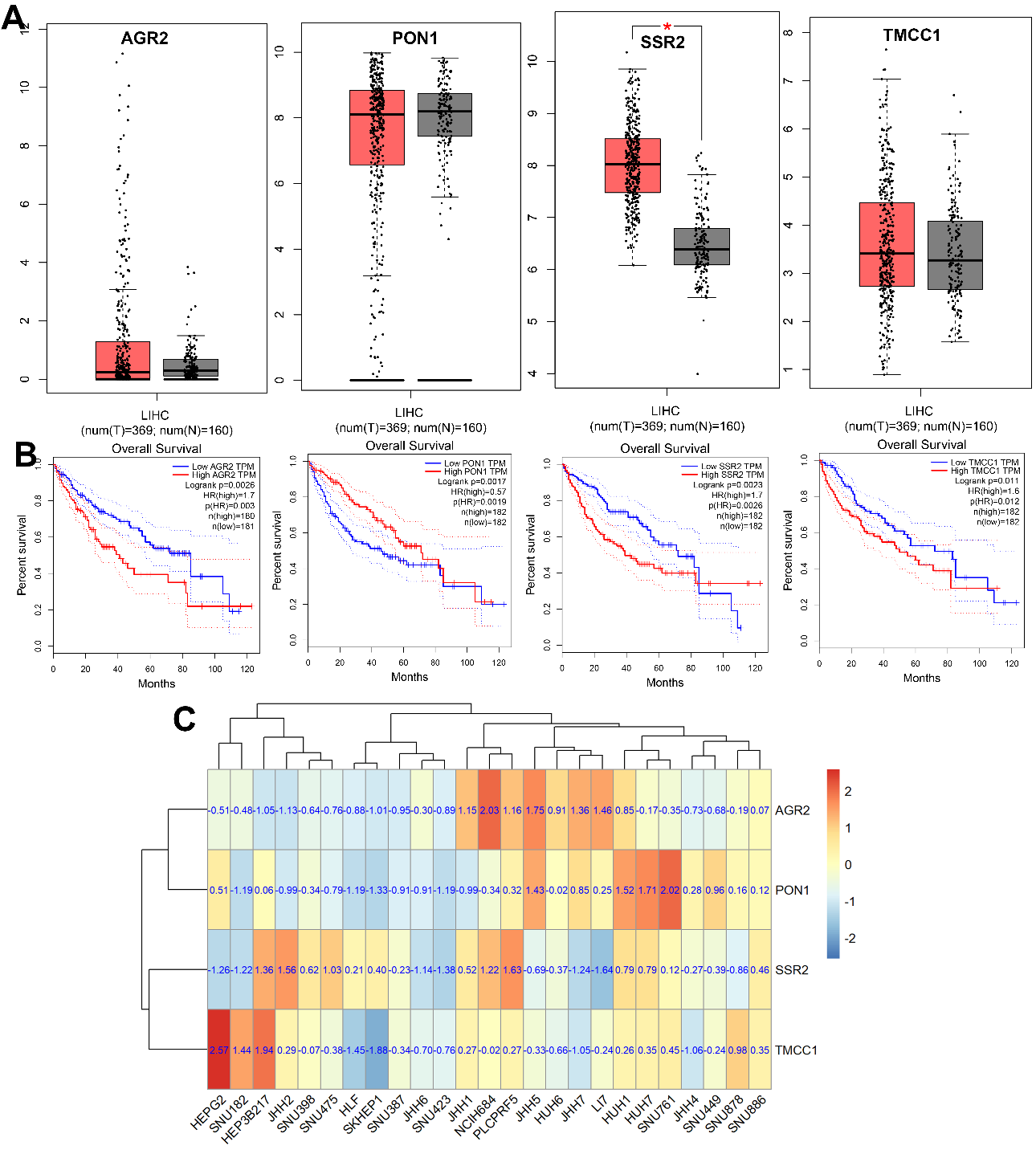


Figure S1 Expression levels and prognostic values of the four genes in normal and HCC tissues explored in GEPIA (A-B) and CCLE database (C).


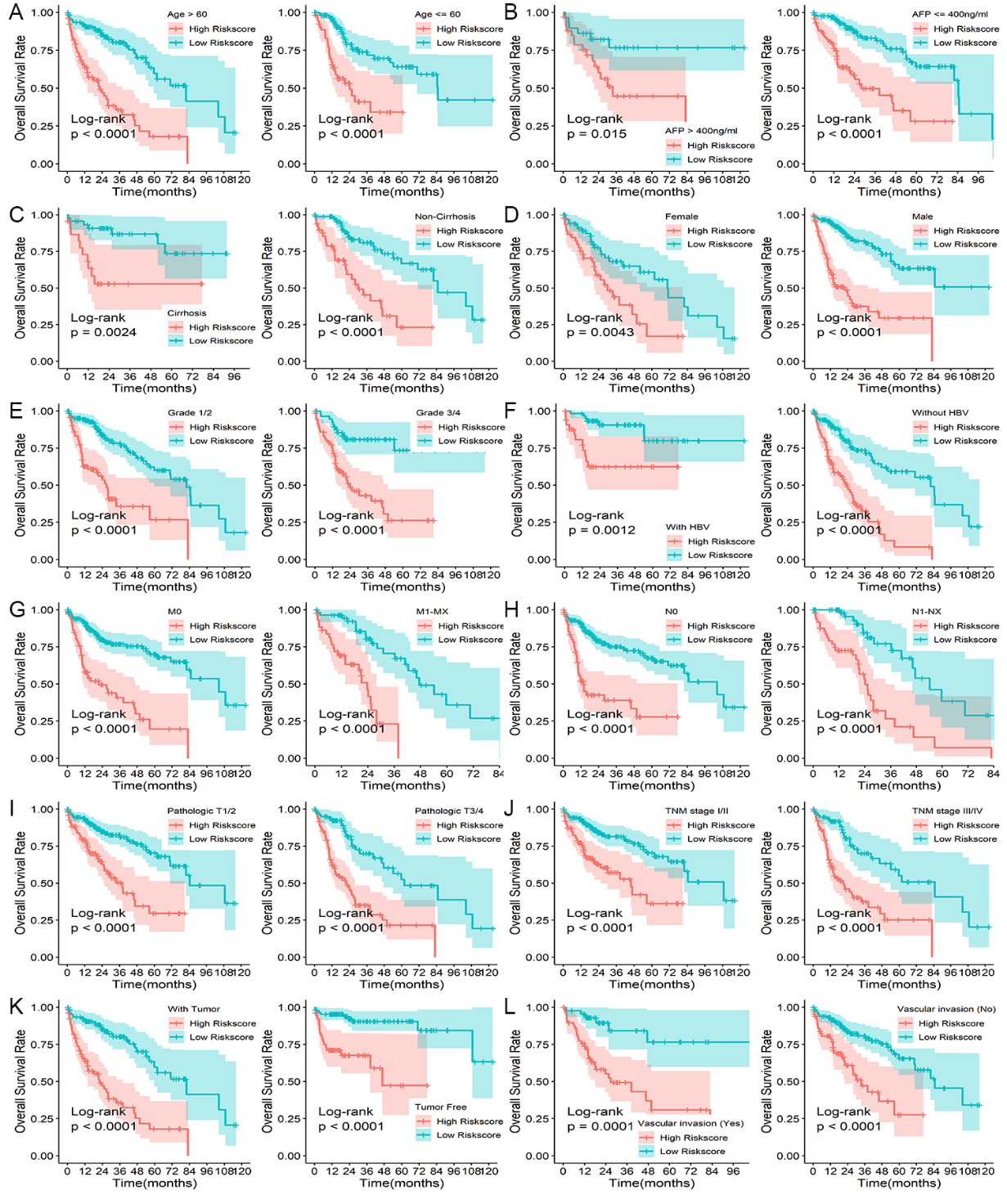


Figure S2 This prognostic model could further differentiate patients with different clinical characteristics.


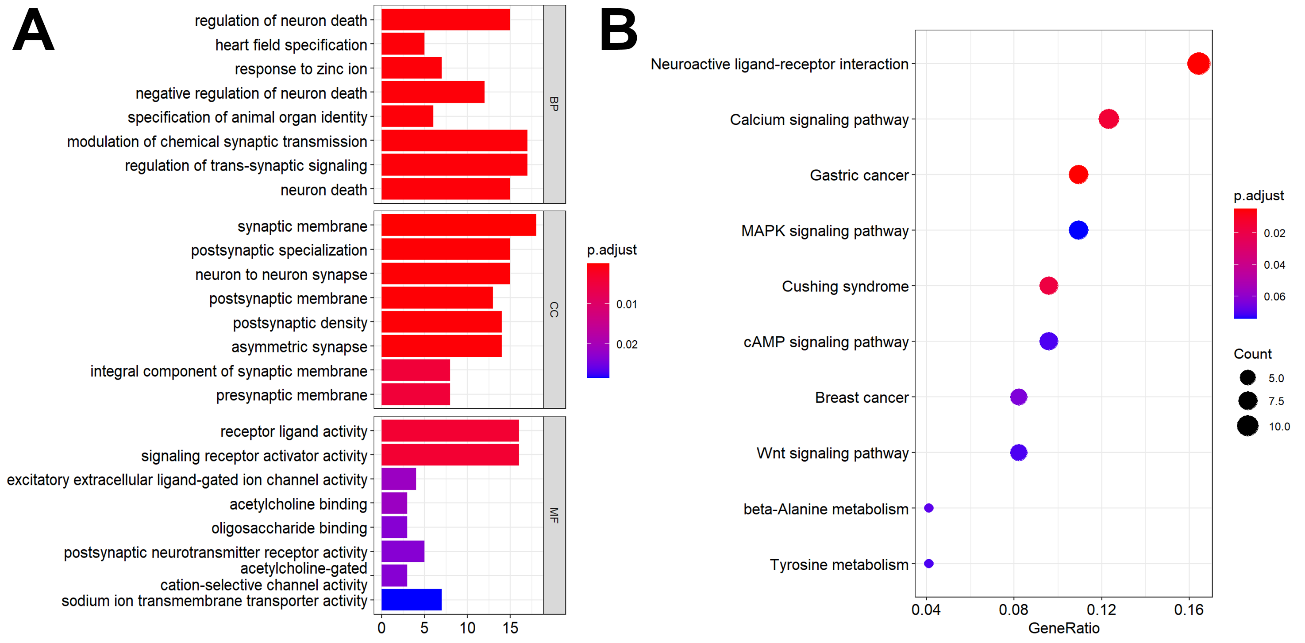


Figure S3 The results of Go and KEGG functional analysis of the differential genes in the high- and low-risk score groups.


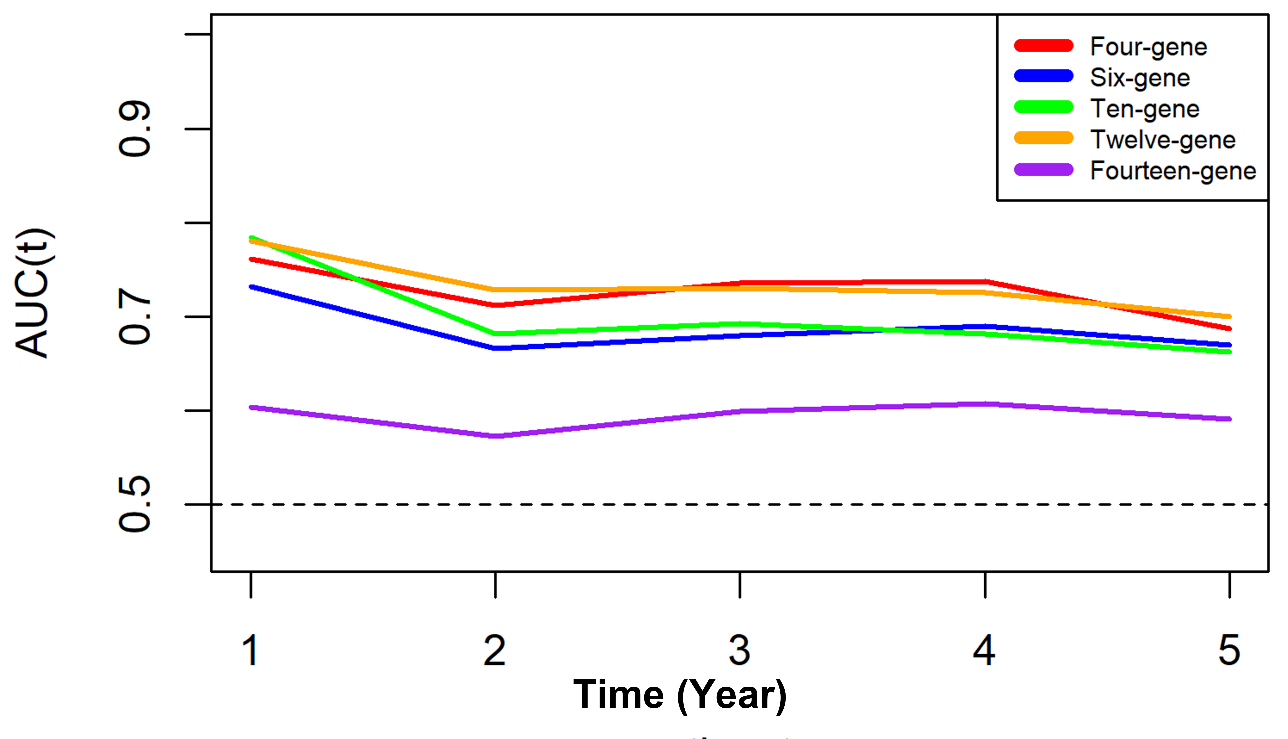


Figure S4 Comparison of the predictive power of the four-gene prognostic model with other prognostic models for survival.


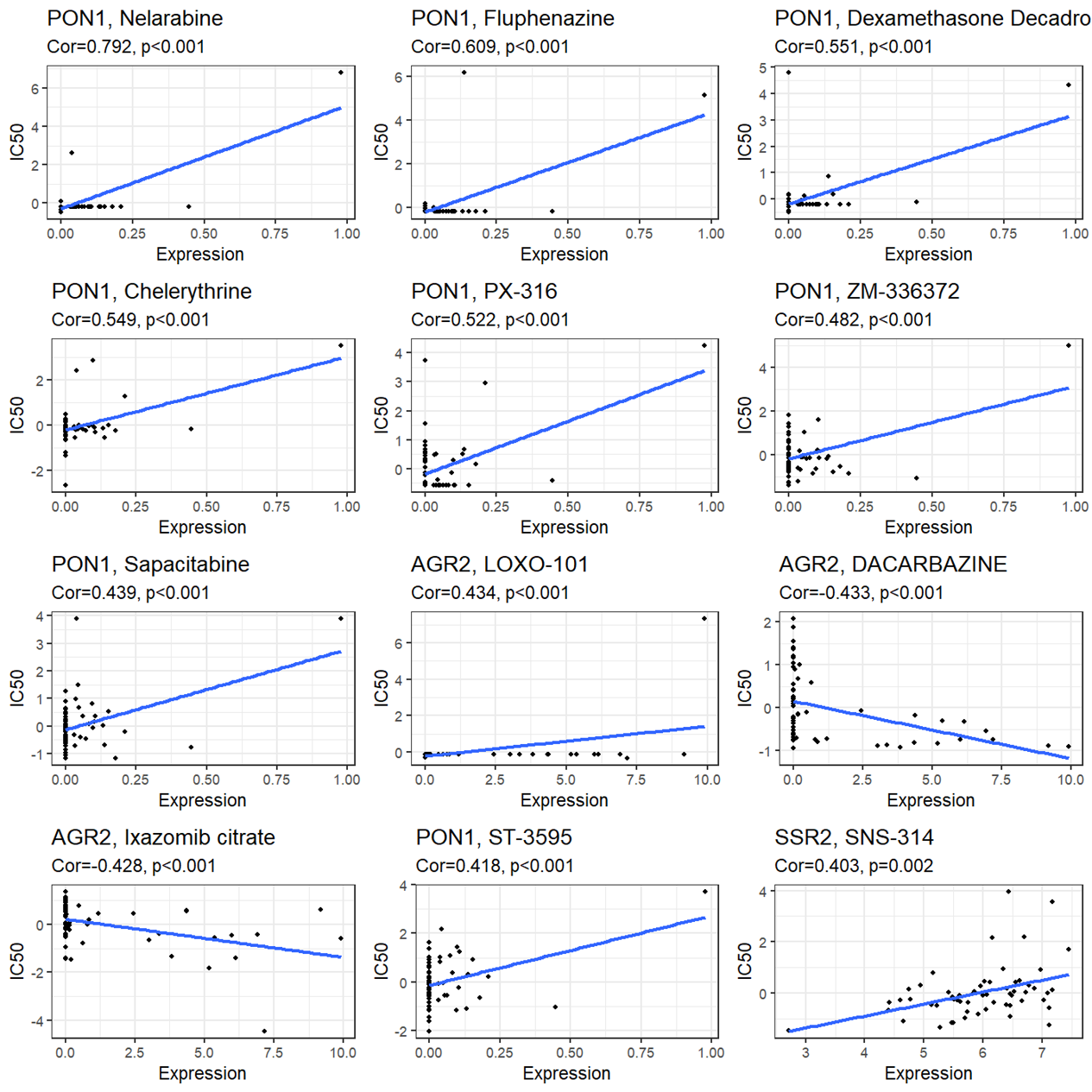


Figure S5 Top 16 most important tumor-sensitive drugs.

Table S1 Clinical characteristics of HCC patients involved in the study

|  | TCGA cohort  (N=365) | ICGC cohort  (N=227) | GSE14520 cohort  (N=216) | Independent cohort  (N=20) |
| --- | --- | --- | --- | --- |
| Gender Male | 119 | 61 | 187 | 15 |
| Female | 246 | 166 | 29 | 5 |
| Age ≤60 years | 173 | 49 | 178 | 7 |
| >60 years | 192 | 178 | 38 | 13 |
| Grade G1/2 | 230 |  |  | 11 |
| G3/4 | 130 |  |  | 9 |
| unknown | 5 |  |  |  |
| TNM Stage I/II | 254 | 140 | 167 | 11 |
| III/IV | 87 | 87 | 49 | 9 |
| unknown | 24 | 0 | 0 |  |
| Vascular Invasion Yes | 106 |  |  |  |
| No | 205 |  |  |  |
| unknown | 5 |  |  |  |
| Recurrence With tumor | 122 |  | 120 |  |
| Tumor free | 161 |  | 96 |  |
| unknown | 82 |  |  |  |
| Cirrhosis With | 68 |  | 198 |  |
| Without | 141 |  | 18 |  |
| unknown | 156 |  | 0 |  |
| HBV or HCV Infection |  |  |  |  |
| Yes | 149 |  | 209 |  |
| No | 203 |  | 6 |  |
| unknown | 13 |  | 1 |  |
| **Child-Pugh A** | 216 |  |  |  |
| B | 21 |  |  |  |
| C | 1 |  |  |  |
| unknown | 127 |  |  |  |

Table. S2 The sequences of the qRT-PCR primers used in this study

| Gene | Forward primer | Reverse primer |
| --- | --- | --- |
| PON1 | CTGATTGCGCTCACCCTCTT | CGGAGAGCATTAAGTCGTGTTTG |
| AGR2 | GTCAGCATTCTTGCTCCTTGT | GGGTCGAGAGTCCTTTGTGTC |
| SSR2 | CTGCTGTCATTTGTGGTGTTG | CTCCACGGCGTATCTGTTCAG |
| TMCC1 | AATCTGCCCAAACTATCCTCCA | CACCTTTGCTCCTACATCCTTC |
| β-ACTIN | CGTGGGCCGCCCTAGGCACCA | TTGGCTTAGGGTTCAGGGGGG |

Table. S3 55 tumor-sensitive drugs targeting tumor cell stemness.

| Gene | Drug | Correlation coefficient | | P value |
| --- | --- | --- | --- | --- |
| PON1 | Nelarabine | | 0.792296 | 7.73E-14 |
| PON1 | Fluphenazine | | 0.609136 | 3.06E-07 |
| PON1 | Dexamethasone Decadron | | 0.551055 | 6.11E-06 |
| PON1 | Chelerythrine | | 0.549238 | 6.65E-06 |
| PON1 | PX-316 | | 0.521619 | 2.27E-05 |
| PON1 | ZM-336372 | | 0.48181 | 0.000112 |
| PON1 | Sapacitabine | | 0.438756 | 0.000509 |
| AGR2 | LOXO-101 | | 0.433822 | 0.000598 |
| AGR2 | DACARBAZINE | | -0.43344 | 0.000605 |
| AGR2 | Ixazomib citrate | | -0.42798 | 0.000721 |
| PON1 | ST-3595 | | 0.41841 | 0.000974 |
| SSR2 | SNS-314 | | 0.403354 | 0.001537 |
| TMCC1 | Raltitrexed | | -0.40207 | 0.001596 |
| TMCC1 | PKM2 (9) | | 0.401362 | 0.001629 |
| TMCC1 | SHP-099 | | 0.401285 | 0.001633 |
| SSR2 | Sabutoclax | | 0.399942 | 0.001699 |
| AGR2 | PD 173074 | | -0.39729 | 0.001835 |
| TMCC1 | XAV-939 | | 0.396379 | 0.001884 |
| TMCC1 | ARRY-380 | | 0.39408 | 0.002013 |
| TMCC1 | PHA-665752 | | -0.39237 | 0.002114 |
| AGR2 | PF-477736 | | -0.38981 | 0.002274 |
| TMCC1 | Bafetinib | | -0.38772 | 0.002413 |
| TMCC1 | EGF-816 | | 0.384443 | 0.002645 |
| AGR2 | Rabusertib | | -0.38331 | 0.00273 |
| AGR2 | KHK-Indazole | | 0.38298 | 0.002755 |
| PON1 | SNS-314 | | 0.381528 | 0.002868 |
| AGR2 | Kahalide F | | 0.371447 | 0.003773 |
| AGR2 | Cisplatin | | -0.37077 | 0.003842 |
| PON1 | Arsenic trioxide | | 0.370096 | 0.003912 |
| TMCC1 | BMS-863233 | | 0.369252 | 0.004001 |
| AGR2 | Ixazomib | | -0.36577 | 0.004387 |
| AGR2 | 3-Bromopyruvate (acid) | | -0.36548 | 0.004421 |
| AGR2 | DMAPT | | -0.36521 | 0.004453 |
| AGR2 | Arsenic trioxide | | -0.36419 | 0.004573 |
| PON1 | Pluripotin | | -0.36385 | 0.004614 |
| TMCC1 | Nilotinib | | -0.36079 | 0.004997 |
| PON1 | Cyclophosphamide | | 0.359811 | 0.005126 |
| PON1 | Fludarabine | | 0.358742 | 0.005269 |
| AGR2 | CCT-245737 | | -0.35664 | 0.005561 |
| TMCC1 | Ibrutinib | | 0.353788 | 0.00598 |
| TMCC1 | Afatinib | | 0.352608 | 0.006162 |
| AGR2 | SGI-1027 | | 0.352604 | 0.006162 |
| SSR2 | salinomycin | | 0.35224 | 0.006219 |
| PON1 | Asparaginase | | 0.350646 | 0.006474 |
| PON1 | entosplenitib | | -0.35024 | 0.00654 |
| SSR2 | AZD-3514 | | 0.348512 | 0.006829 |
| SSR2 | Fenretinide | | 0.347266 | 0.007044 |
| PON1 | Calusterone | | 0.346611 | 0.00716 |
| PON1 | Dexrazoxane | | 0.344669 | 0.007512 |
| SSR2 | Quizartinib | | 0.344365 | 0.007568 |
| TMCC1 | ARRY-614 | | -0.34229 | 0.007963 |
| TMCC1 | Lenvatinib | | 0.338534 | 0.008726 |
| PON1 | Navitoclax | | 0.334292 | 0.009661 |
| AGR2 | IDH-C227 | | -0.33387 | 0.00976 |
| TMCC1 | AZ-628 | | -0.33334 | 0.009883 |
